# Proximal contact alterations between implant-supported restorations and adjacent teeth in the posterior region: A 3-month prospective study

**DOI:** 10.4317/jced.57802

**Published:** 2021-05-01

**Authors:** Solange Mehanna, Pascale Habre-Hallage

**Affiliations:** 1 DDS, MSc. Clinical Instructor, Department of Prosthodontics, Faculty of Dental Medicine, Saint Joseph University, Beirut, Lebanon; 2 DDS, MSc, PhD. Professor, Director of the post-graduate program of fixed prosthodontics, Former Head of the Department of Prosthodontics, Faculty of Dental Medicine, Saint Joseph University, Beirut, Lebanon

## Abstract

**Background:**

Interproximal contact loss (ICL) is a multifactorial implant complication. The aims of this prospective clinical study were to evaluate proximal contact alterations between implant-supported fixed prostheses (IFPs) and adjacent teeth and to identify potential contributing factors.

**Material and Methods:**

This study was conducted from April to September 2019 at the Department of Prosthodontics. Forty-three patients (23 females and 20 males, age range 31-70) treated with 43 posterior IFPs were recruited for this study. All proximal contacts (64) were checked visually and radiographically. Proximal contact tightness (PCT) was clinically evaluated using dental floss and measured during removal of a calibrated 0.05 mm thick metal strip previously inserted into the proximal area. Mesial and distal PCT were measured at restoration insertion (T0), 1-month follow-up (T1), and 3-month follow-up (T2). ICL was assessed in relation to the patients’ age, gender, smoking habits, implant system, proximal contact position, jaw position and restoration type of the implant prostheses. The significance level was set at P value ≤ 0.05.

**Results:**

The PCTs between fixed implant prostheses and adjacent teeth decreased significantly between T0 and T2. Restoration type affected the loss of interproximal contact at the mesial (free-end restorations; *P* = 0.008) and distal aspects (*P*< 0.001), whereas implant system affected only the distal aspects of the proximal contacts (*P* = 0.002).

**Conclusions:**

Proximal contact tightness between fixed implant prostheses and adjacent teeth decreased over the 3-month observation period. Contact loss between fixed implant prostheses and adjacent teeth may be influenced by restoration type and implant system.

** Key words:**Adjacent teeth, dental implants, implant complication, implant fixed prostheses, interproximal contact loss, proximal contact strength, proximal contact tightness.

## Introduction

It is undeniable that the advent of osseointegration has had a fundamental impact on therapeutic approaches and strategies implemented today in the field of prosthetic rehabilitation (1). In fact, osseointegrated dental implants have been a successful treatment modality in partial and complete edentulism for more than 35 years (2). Although high survival rates were reported, 95% and 86.7% after 5- and 10-years respectively (3), a wide variety of biological, technical, and aesthetic complications have been extensively documented (4). Over the last decade, interproximal contact loss (ICL) between implant-supported fixed prostheses (IFPs) and adjacent teeth has been increasingly reported as a complication in daily clinical practice (2,3,5-13). While acknowledging the fact that implants lack the inherent physiological mobility of teeth (14), proximal contact tightness (PCT) becomes more critical in implant-supported prostheses (15).

ICL can be defined as the absence of interproximal contact between implant prostheses and adjacent teeth, where initially firm contact was established by the clinician (16).

Several studies have investigated the occurrence of open contacts that developed after implant restorations were inserted next to teeth. According to these studies, the prevalence of ICL varied between 17% and 66% (2,3,5-13). This unpredictable complication occurred as early as 3 months after prosthetic rehabilitation, usually on the mesial aspect of an implant restoration and posterior in the arch (17).

As the phenomenon of ICL seems to be multifactorial (16), several authors have attempted to identify possible causative factors. The aims of this prospective clinical study were to evaluate proximal contact alterations between implant-supported fixed prostheses and adjacent teeth and to identify potential contributing factors. The null hypothesis was that the proximal contact strength does not undergo significant changes over the 3-month observation period.

## Material and Methods

-Ethic statement 

The study protocol was submitted to the Ethical Committee of Scientific Research of Saint Joseph University in Beirut, Lebanon (USJ-2020-02). All procedures were conducted in accordance with approved guidelines and regulations. All patients were informed that the results of interproximal contact tightness would be used in a clinical study. Before enrolment, informed consent was obtained from all subjects.

-Selection of study sample

This clinical prospective study was conducted from April to September 2019 at the Department of Prosthodontics, Faculty of Dental Medicine, Saint Joseph University, Beirut.

Patients who had been treated with implant-supported single crowns (SC) or implant-supported fixed partial dentures (FPDs), in the posterior region, were screened during the period of data collection.

Subjects were included in the study if they met the following criteria: (i) presence of complete permanent dentition (with or without the third molars), (ii) implants placed in the posterior area distal to the canine teeth, (iii) adjacent and opposing dentition involving natural or restored teeth and single crowns delivered on natural teeth, (iv) no signs or symptoms of food impaction, periodontal disease or temporomandibular disorders and (v) periodic follow-up record. On the other hand, participants were excluded due to the following: (i) systemic disease such as bone or metabolic disease, (ii) a history of orthodontic therapy or periodontal surgery, (iii) adjacent natural teeth or fixed dental prostheses with pathological mobility (≥ 1 mm), (iv) parafunctional habits and (v) adjacent restorations after prostheses delivery.

In accordance with the selection criteria, 43 participants (20 men and 23 women) between 31 and 70 years of age (51.72 ± 9.540) were enrolled in this study. Among the 43 IFPs supported by 64 implants, 23 IFPs were supported by a single implant, 19 IFPs by two implants and 1 IFP by three implants; 15 IFPs were in the maxilla and 28 IFPs in the mandible. A total of 64 interproximal contacts between the implant-supported prostheses and adjacent teeth were evaluated up to 3 months after IFP insertion. The subject characteristics are presented in Table 1.

**Table 1:**
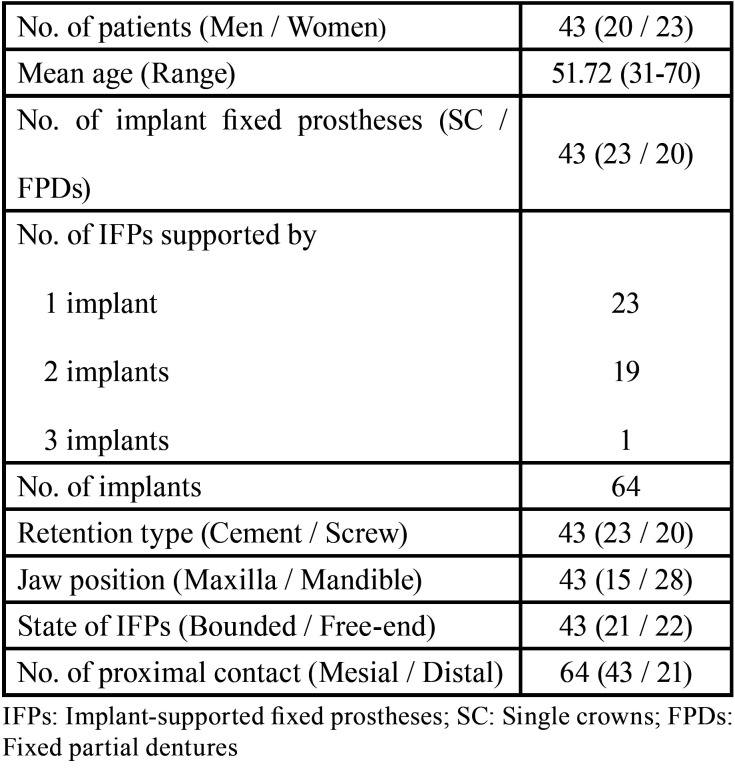
Description of the study samples.

-Implants and prostheses

All surgical and restorative phases were performed by post-graduate residents in the Departments of Implantology and Prosthodontics, Saint Joseph University. All implants were placed using a surgical guide provided by the prosthodontist. The implant systems used in this study were Shelta (Sweden & Martina), Replace (Nobel Biocare), Straumann (Straumann), 3I (Biomet), Zimmer (Biomet), Klockner (Klockner) and Astra (Dentsply Sirona), in order of frequency of use. The definitive implant prostheses were single crowns or fixed partial dentures, which consisted of porcelain-fused-to-metal crowns or zirconia crowns. The implants were placed at bone or tissue level and the prostheses were either cemented or screw retained.

Before IFPs insertion, all proximal and occlusal contacts were checked. The proximal contacts were adjusted so that waxed dental floss (Oral-B®, Essential floss 70 μm) could be inserted with definite resistance. Furthermore, occlusal contacts were evaluated with 8 μm shim stock according to the implant protective occlusion concept. After prosthesis delivery, proximal contacts were evaluated by the post-graduate residents, approved by the fellow clinician, and then assessed by the investigator.

All cement-retained IFPs were delivered with Temp-Bond™ (Kerr Corporation), a non-eugenol temporary cement, to secure the retrievability of IFPs. Screw-retained IFPs were secured according to the manufacturer’s instructions and retightened 3 days later.

-Clinical assessments

Before taking any measurements, each participant was thoroughly examined by the investigator to ensure that the previously mentioned inclusion criteria were satisfied. All clinical assessments were recorded at baseline (immediately after prosthesis delivery; T0) and at follow-up visits conducted 1 month (T1) and 3 months (T2) after functional loading.

The proximal contact tightness was visually and radiographically inspected, evaluated using dental floss and measured based on the frictional force concept, which occurs during removal of the metal strip. In addition, patients were asked if there was any discomfort related to food impaction.

The measurement of PCT was carried out directly in the patients’ mouths. Each participant was seated in a dental chair in a reclined position, at 45 degrees, with head support. The proximal contact area was dried with a gentle air stream before the measurement. All measurements were operated at rest state and the subjects were restricted not to occlude during the measuring procedure. The recordings were performed by the same investigator for standardization.

Waxed dental floss was used to evaluate the degree of proximal contact tightness between implant-supported prostheses and adjacent teeth. If the dental floss could be inserted into an interproximal contact with definite resistance, the tightness of the proximal contact was regarded as “tight”; it was deemed as “loose” or “open” if the dental floss could be inserted with minimal or without resistance (ICL).

In addition, the PCT between implant prostheses and adjacent teeth was measured by an orthodontic dynamometer (Richmond orthodontic stress and tension gauge, ETM Corporation).

**Figure 1:**
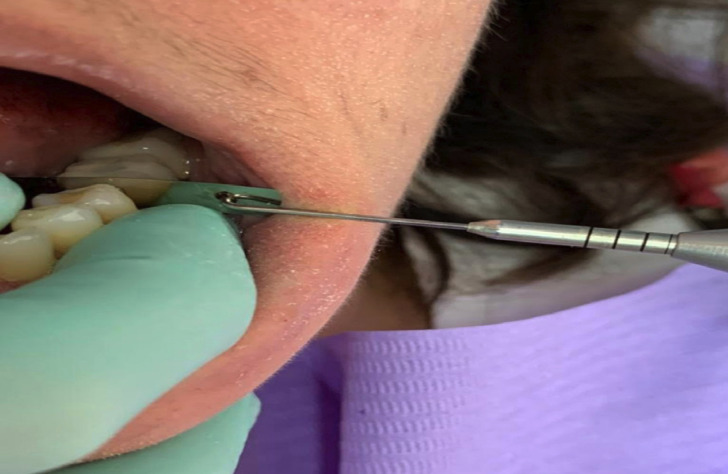
Frictional force measurement.

A Tofflemire® Matrix, 5 mm wide, 25 mm long and 0.05 mm thick, was inserted into the proximal area along a vertical pathway (perpendicular to the occlusal plane) and hooked to the measuring gauge. When the metal strip was slowly removed in a bucco-lingual direction (Fig. 1), a frictional force occurred. The proximal contact tightness was quantified as the maximum frictional force. Visible deformations of the metal strip during positioning led to a repetition of the experiment. This gauge could measure up to 4.45 N.

Both mesial and distal PCT were measured in bounded implant-supported prostheses, whereas only mesial contact was recorded for free-end prostheses. In each session, proximal contact tightness was repeated three times, with at least 2-minute rest interval, to ensure validity. The mean value was determined and recorded for analysis.

-Radiographic assessment 

Upon insertion of the prostheses and at recall examination, periapical radiographs were taken with a digital X-ray sensor parallel and an X-ray beam perpendicular to the proximal embrasure between the implant-supported prosthesis and the tooth.

-Patient assessment 

At the recall examination, the patients were asked to report whether they had experienced food impaction in the proximal embrasure between the teeth and IFPs. If food impaction was noted, the patient was asked to locate the food impacted embrasure, i.e., mesial, distal or both in relation to the implant-supported prosthesis (patient awareness).

-Study variables 

The variables including patients’ age, gender, smoking habits, implant system, proximal contact position (mesial or distal), jaw position (maxilla or mandible), restoration type (SC or FDPs; cement or screw-retained) were collected on a structured proforma.

-Statistical analysis

Statistical analyses were performed using a software program SPSS for Windows version 25.0 (Chicago, IL, USA). The alpha error was set at 0.05. The Kolmogorov-Smirnov test was used to verify the normality distribution of continuous variables. Repeated measures analyses of variance followed by Bonferroni multiple comparisons were conducted to compare the force within time for mesial and distal contact.

Student t test and Mann-Whitney test were performed to compare continuous variables between two groups. ANOVA followed by Bonferroni multiple comparisons and Kruskal-Wallis tests were performed to compare continuous variables between three or more groups. Fisher Exact test was performed to compare percentage. The relationship between continuous variables were assessed using Pearson correlation coefficient.

## Results

Data from 43 mesial contact areas and 21 distal contact areas between implant restorations and adjacent teeth were analyzed. 95.3% of the mesial contact areas evaluated with floss and 90.5% of the distal contact areas remained tight at the 3-month follow-up visits (Table 2). The difference was not statistically significant (*P* = 0.592).

**Table 2:**
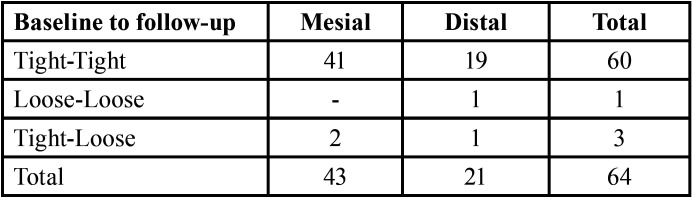
Floss evaluation of proximal contacts over time.

The mean and the standard deviation of the proximal contact strength are shown in Table 3.

**Table 3:**
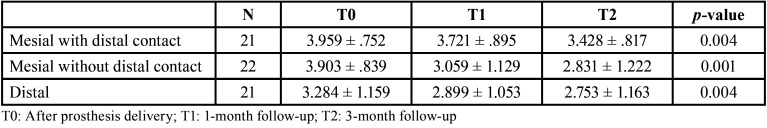
Quantitative evaluation of proximal contact strength (Newton).

The strength of mesial contact areas with distal contacts decreased significantly over time (*P* = 0.004); the difference was not significant between T0 and T1; the force significantly decreased at T2 (*P* value = 0.009).

The strength of mesial contact areas without distal contacts decreased significantly over time (*P* < 0.001); the force significantly decreased between T0 and T1 (*P* value < 0.001); the difference was not significant between T1 and T2 (*P* = 0.080).

The strength of distal contact areas significantly decreased over time (*P* = 0.004); this force was highest in T0, intermediate in T1 and smallest in T2.

The decrease of the force of mesial contacts in the absence of distal contacts within time was elevated (1.072) (*P* value = 0.029). The decrease of the force of mesial contacts in presence of distal contacts within time (0.531 N) was not significantly different from the decrease of the force of distal contacts (0.531 N) (*P* > 0.05).

-Factors associated with proximal contact strength 

The age of the participants was not significantly correlated with the strength of mesial contact areas in presence of distal contacts (*P* = 0.844). In addition, gender (*P* = 0.635), implant system (*P* = 0.670), type of restoration (*P* = 0.557) and jaw position (*P* = 0.116) did not affect the strength of the contact areas (Table 4).

**Table 4:**
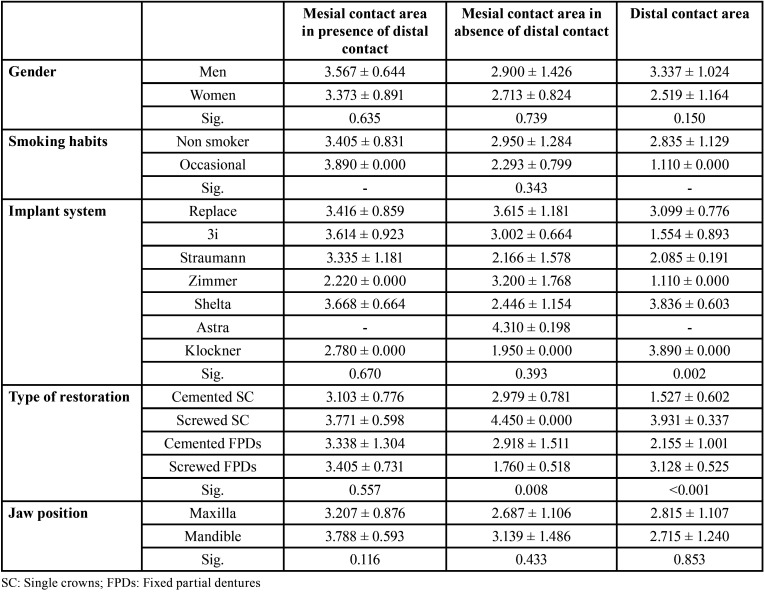
Factors associated with the strength of the contact area over time.

The age of the participants was not significantly correlated with the strength of mesial contact areas in absence of distal contacts (*P* = 0.844). In addition, gender (*P* = 0.739), smoking habits (*P* = 0.343), implant system (*P* = 0.393) and jaw position (*P* = 0.433) did not affect the strength of mesial contact areas. On the other hand, the type of prosthetic restoration affected significantly the contact areas; it was highest in single screwed crowns (*P* = 0.008) (Table 4).

Age (*P* = 0.427), gender (*P* = 0.150), smoking habits and implant site (*P* = 0.853) did not affect the strength of distal contact areas. The type of prosthetic restoration affected significantly the contact areas; it was highest in single screwed crowns. The implant system significantly affected the distal contact areas (*P* < 0.001); the strength was highest at the Klockner and Shelta implants, and lowest at the 3i and Zimmer implants (Table 4).

-Inspection tool accuracy 

General linear model performed was used to study the difference between the scores obtained using 6 inspection tools. The scores were unified under the scale of good resembled by 2 and bad resembled by one. For qualitative variables, the proximal contacts were assessed and dichotomized as “present” or “absent”. However, for quantitative variable, the dichotomization process was based on the mean value.

**Figure 2:**
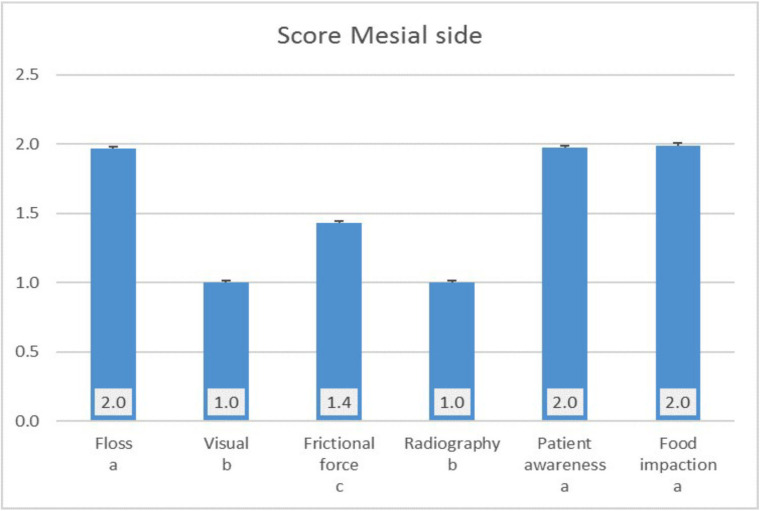
Score of the mesial side.

**Figure 3:**
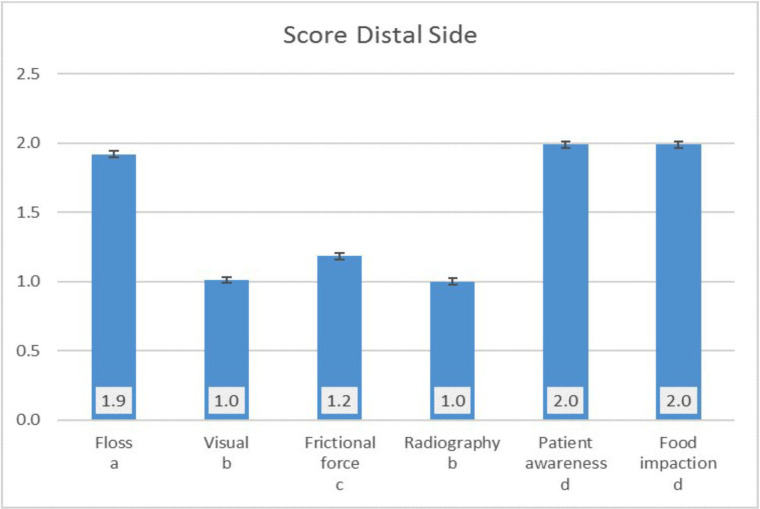
Score of the distal side.

Six-inspection tools were applied to evaluate the score of the implant. The score of the inspection tools for both mesial and distal side was significantly the lowest for the visual and radiography method, followed by the frictional force method which was significantly lower than the score obtained using floss, patient awareness and food impaction (Figs. 2,3).

## Discussion

Interproximal contact loss, between fixed implant-supported restorations and adjacent teeth has been a complication in implant dentistry (18). It has been speculated that proximal contact loss is an unpredicTable multifactorial phenomenon which may occur over months or years after implant prosthesis delivery (16,17).

The incidence of interproximal contact loss varied between 17% to 66% over a follow-up period ranging from 1 to 156 months (2,3,5-13). Nonetheless, this heterogeneity among studies may be due to patient’s age, study population, prosthesis design, types of adjacent restorations, occlusal forces, opposing dentition, monitoring period, different methodologies to assess the integrity of the interproximal areas and different statistical analyses (17).

Although several studies addressed the incidence of ICL, only one study distinguished between the development of open contacts (floss passes between teeth unimpeded) and the creation of loose contacts (floss encounters weak resistance) (6). Furthermore, previous studies noted that the earliest occurrence of open contacts, between IFPs and adjacent teeth, was detected after 3, 6 and 8 months of function (5,6,10).

Interestingly, the earliest occurrence of ICL was noted after 3 months (5), therefore the present investigation targeted the proximal contact alterations during a 3-month observation period.

At the time of IFP delivery, a tight proximal contact was established across all proximal contacts. Although different methods have been used to evaluate the proximal contact strength (PCS), there was no consensus regarding the most accurate technique. The PCS was evaluated, in the literature, by the ease of passing of dental floss, various thicknesses of shim stocks, metal strips insertion, and tooth pressure meter (19). Consequently, PCS was clinically evaluated in this study using a dental floss and quantified by measuring the frictional force which occurred when the metal strip was removed from the proximal area. It must be noted that this measuring technique has some limitations in interpreting the PCS, as it alters the physiological arrangement (20). In addition, the interproximal areas were checked visually and radiographically. Nevertheless, patient awareness and assessment in case of food impaction were also considered as an inspection tool. Among these inspection tools, it has been shown that the frictional force assessment remains the most objective therefore the most accurate technique.

Osborn (21) was the first to construct a device for PCT measurements based on the frictional force concept. However, several modifications of this device were described in the literature. Given that a thicker strip would give rise to a subtle change and a thinner strip could be easily torn intraorally, a strip of 0.05 mm thickness was chosen. Additionally, a rest interval was ensured between measurements to allow a physiological recovery of the proximal area.

The original proximal contact strength was lost, to some extent, during the follow-up visits. Therefore, the null hypothesis was rejected as significant differences in PCTs were found over time. The present finding is in agreement with a previous study (15). In fact, Ren *et al.* (15) investigated consecutive changes in PCT and concluded that PCT decreased significantly over the 3-month observation period after IFP delivery. However, the deliberate increase in the PCT was unsTable and diminished in less than 3 months. It is worth mentioning that six ICLs occurred within 1 month and were excluded from the data analyses (9). Intriguingly, Zeng *et al.* (22) also noted the first open contact 1-month after crown insertion.

However, the results of this study might imply that a slightly tighter proximal contact, upon insertion, may delay the proximal contact loss phenomenon. This assumption is supported by the study of Shi *et al.* (13) However, this statement should be confirmed by future studies with longer follow-up periods.

Considering proximal contact position, ICL may develop at the mesial as well as the distal aspect of the proximal contact owing to the positional changes of the adjacent teeth in relation to the IFP (6). In the present study, both mesial and distal PCTs decreased significantly throughout the follow-up period. A power test, for the comparison within time between mesial and distal, was calculated and was found to be greater than 80%.

This analysis indicated that the mesial aspects were more likely to be lost than the distal ones, which agrees with previous reports (2,6,9,12). The free-end restorations showed the highest contact loss throughout the assessment period which coincides with the findings of Luo *et al.* (8) who stated that the contact loss in the free-end prosthesis was 2.870 times that of bounded restorations. Conversely, the ICL was not influenced by the proximal contact position (mesial or distal) in the analysis of 21 IFPs that were bordered by both mesial and distal teeth. This could be attributed to a relatively short observation period (3-month).

On one hand, restoration type influenced the PCS of the mesial contact (free-end restorations) and the distal contact. It was reported that various rotational freedoms of butt joint implant-abutment components and uncontrollable vertical positioning of conical abutment-implant assemblies may lead to contact discrepancies at delivery. Also, conical abutment settling may increase the PCT over time (16). Owing to these reasons, the highest PCT was noted in the single screwed restorations. Moreover, the implant system influenced only the PCS of the distal contact. This could be attributed to the connection design, number of implants and higher occlusal load in the distal region. However, these findings should be interpreted with caution until confirmed by future studies.

On the other hand, patients’ gender, smoking habits and arch position did not significantly influence the proximal contact loss. Regarding gender, the result of the present study corroborates with previous reports (2,6,8-10,12,13). As far as arch position is concerned, this finding is in line with previously published studies (6,8,10).

The present study revealed some shortcomings. First, different implant systems and different prostheses material were included. In addition, PCTs in other quadrants were not used as control groups. Another limitation was the uncontrolled removal speed of the metal strip as well as different prosthesis materials which might affect the PCS. Finally, the follow-up period was relatively short.

To confirm the present conclusions, studies with longer follow-up period are needed. The results of the current study validate the urge to inform the patients regarding the ICL complication. As the ICL incidence supposedly tends to increase over time, an effort toward the prevention and management of ICL warrants further innovation and investigation.
